# *Morus alba* L. Leaves (WML) Modulate Sweet (TAS1R) and Bitter (TAS2R) Taste in the Studies on Human Receptors – A New Perspective on the Utilization of White Mulberry Leaves in Food Production?

**DOI:** 10.1007/s11130-023-01107-0

**Published:** 2023-10-05

**Authors:** Monika Przeor, Maria Jokiel

**Affiliations:** 1 Department of Gastronomy Science and Functional Foods, Poznań University of Life Sciences, Poznań, Poland; 2 PORT, Polish Center for Technology Development, Wrocław, Poland

**Keywords:** *Morus alba*, TAS2R receptors, Bitter taste, Taste modulator, White mulberry

## Abstract

From the nutritional perspective, the main direction of the utilization of white mulberry (*Morus alba* L.) parts so far has been to produce dietary supplements or functional foods for individuals with diabetes or over-weight. Its leaves are widely known as a valuable source of bioactive compounds responsible for its antioxidant and antidiabetic effects, both in animals and humans. The authors found that processed leaves can also be investigated as potential bitter and/or sweet taste modulators—an important property of new functional foods. The study aimed to validate the inhibitory effect of *Morus alba* L. on the TAS2R3 and TAS2R13 bitter taste and TSA1R2/TSA1R3 receptors and determine the changes that the conditioning process caused in such receptors. The effect on the receptors was evaluated in specially transfected HEK293T cells, and the inhibition ratio was measured using the calcium release test. Moreover, the stability of phenolics in the simulated intestinal in vitro digestion process was determined. Results showed that the *Morus alba* leaf extracts were rich in gallic, chlorogenic and caffeic acids together with rutin and quercetin 3-(6-malonyl)-glucoside, while the conditioning process positively affected their amount. Most identified phenolics were reduced during in vitro digestion. In the taste receptors test, it was found that the phytochemicals from conditioned *Morus alba* leaf extract enhanced sweet taste, together with a reduction of bitter taste receptor activity in some cases. To conclude, the study has found that *Morus alba*, especially when conditioned for 4 h, seems to be a valuable modulator of taste, which should be considered in future research as a crucial reason for its new utilization.

**Supplementary Information:**

The online version contains supplementary material available at 10.1007/s11130-023-01107-0.

## Introduction

In the past decade, much research has focused on the antioxidant status of white mulberry leaves, related to antidiabetic, anticholesterolemic or anti-obesity activity. *Morus alba* L. parts have been analyzed because of their utilization for nourishing blood, improving kidney function, and treating other various ailments [1–7]. In all its morphological parts a lot of nutrients where found, such as chalcones, coumarins, flavonoids, phenolic acids, stilbens, catechins, aldehydes, terpenoids, steroids, alkaloids, etc. [8, 9]. There are investigations on white mulberry usage as a bioactive component of dietary supplements, as well as food products. The vast majority of them involved mulberry trees cultivated in China, Korea and India [10]. Molecularly, much attention is currently being paid to the effects of active compounds in the context of normal glycemia. Retinol-binding protein 4 and haptoglobinone downregulation was shown as one of the mechanisms responsible for prevention in diabetic patients [11].

All of these areas are undeniably relevant for society, in the era of galloping diabetes incidences. Nevertheless, having the above in mind, it remains unclear what direction can be further taken for a broader or alternative application of the potential of the white mulberry, including the technological aspects—so important in effective food production.

In the area of food sciences, technologists together with nutritionists found that the modulation of bitter taste is important for consumers when introducing some nutritional food products for example containing dark cocoa, medicines, and dietary supplements into the diet. Moreover, it can be a valuable aspect of newly designed functional foods. From a nutritional point of view, a pleasant taste increases the probability that patients will incorporate a health-promoting product into their diet. From the consumers point of view, favorable taste helps break down the natural fear barrier to novelty.

In diet-related diseases, such as overweight, diabetes mellitus, etc., the use of such plant-origin ingredients is often combined with the addition of sugar or sugar substitutes, in order to obtain a functional product with adequate, acceptable palatability. The simplest way for manufacturers seems to be the usage of synthetic or semisynthetic, mostly cheap, sweeteners. However, their safety for sensitive consumers (e.g., children, the elderly, pregnant women) may be questioned from a health standpoint [12]. The search for molecular inhibitors of bitter taste receptors, especially from natural sources such as high-nutritional plants [13–15], is a new and interesting way to improve the quality of many newly created food products for such consumers. A bitter taste may appear in the product due some specific components (some bitter raw materials in functional foods, supplements), as a result of the hydrolysis of some proteins (e.g., soybean) to bitter peptides, microbial activity during fermentation, the effect of very high temperatures during processing, etc. Generally, bitterness is perceived as a result of the association of bitter compounds with specific bitter receptors, which activate signals (mediated by G protein) that reach the brain in the form of electrical signals [16, 17].

It has been scientifically proven, that natural flavonols may inhibit such perception of bitter taste in food [18]. Therefore, in this study *Morus alba* is investigated as a plant exhibiting such properties. It has already been concluded that the composition of biological compounds, including flavonols, in white mulberry leaves is changed or improved as a result of some technological processes [2, 19, 20]. Therefore, the key objective of this study was to assess the overall impact of Polish *Morus alba* leaves (conditioned and non-conditioned) on the activity of taste receptors (masking the bitter taste and enhancing the perception of sweetness). The additional objective was to check whether the valuable components (phenolic acids and flavonols) from *Morus alba* are stable after the simulated in vitro digestion process (Supplementary Material 1). So far, no research has been conducted on taste modulation in the presence of white mulberry leaves in any form.

## Materials and Methods

(Supplementary Material 2).

## Results and Discussion

### Effect of *Morus Alba* Leaves on Taste TAS2R Receptors

**Fig. 1:**
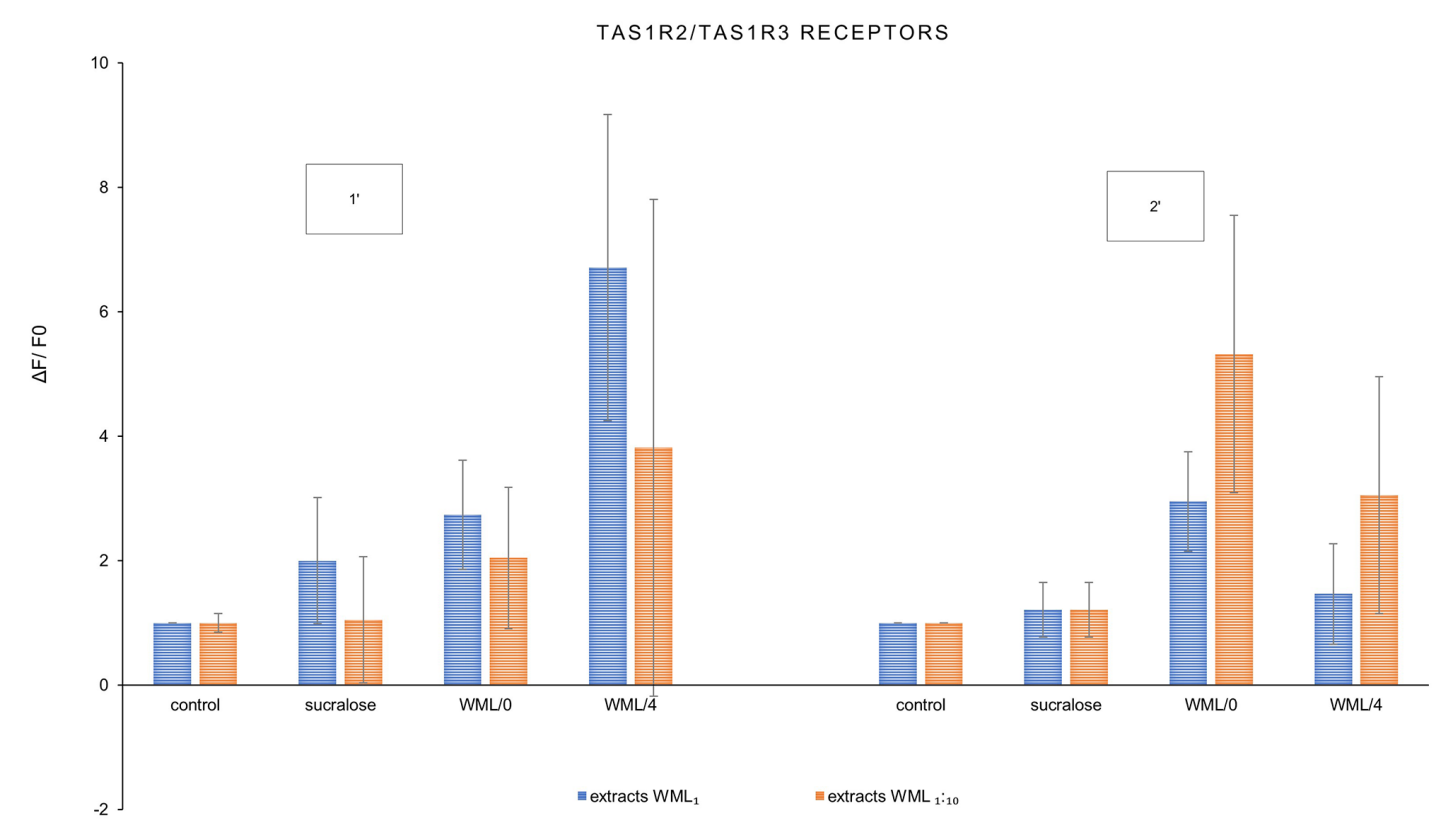
Changes in fluorescence (ΔF/F0) as a response of the TAS1R2/TAS1R3 sweet taste receptors induced by *Morus alba* L. leaf extracts (WML_1_—not diluted, WML_1:10_—diluted 10-fold) and reference sucralose after 1 min [1’] and 2 min [2’] of interaction; n = 3

**Fig. 2:**
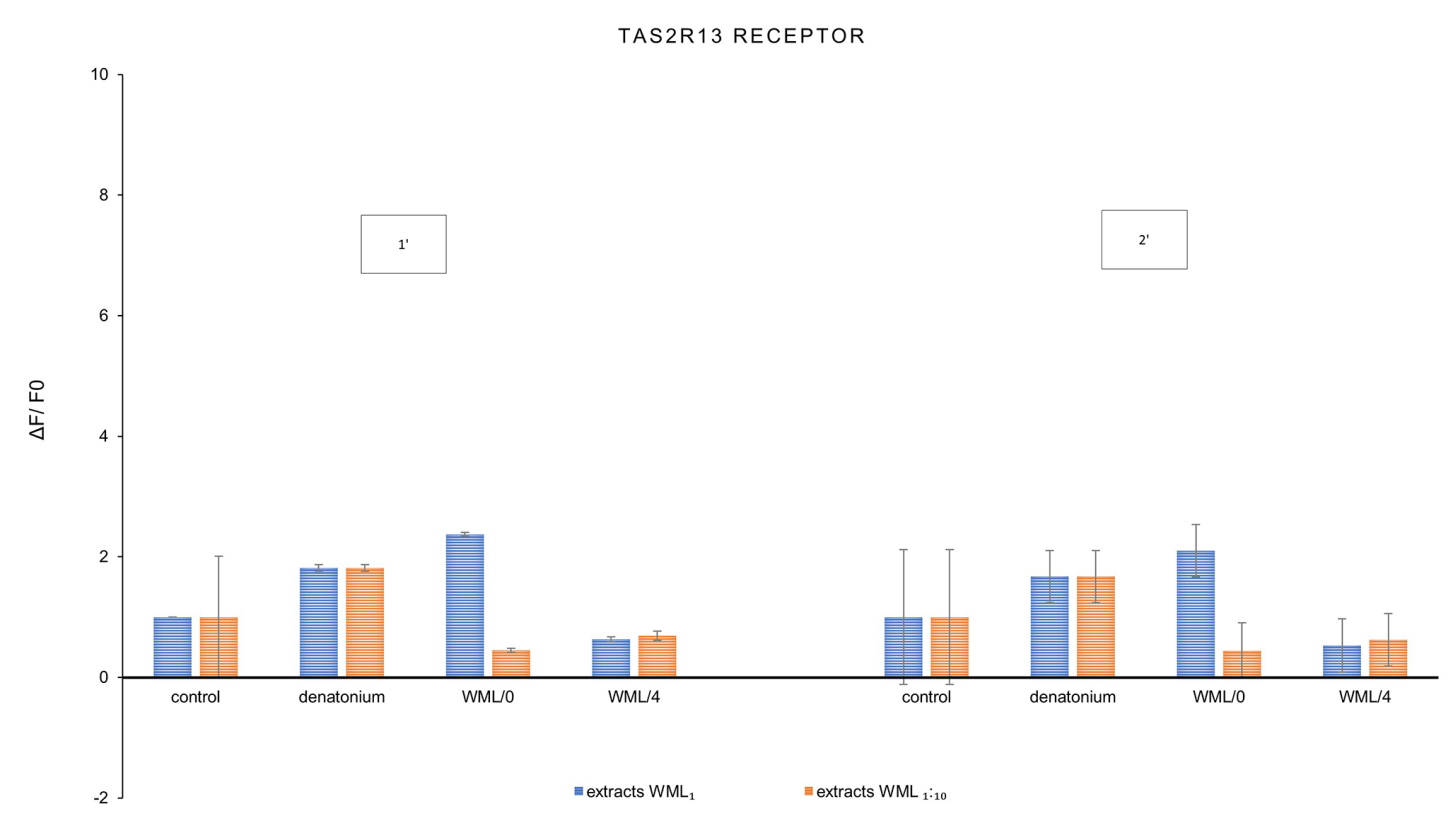
Changes in fluorescence (ΔF/F0) as a response of the TAS2R13 bitter taste receptor induced by *Morus alba* L. leaf extracts (WML_1_—not diluted, WML_1:10_—diluted 10-fold) and reference denatonium after 1 min [1’] and 2 min [2’] of interaction; n = 3

**Fig. 3:**
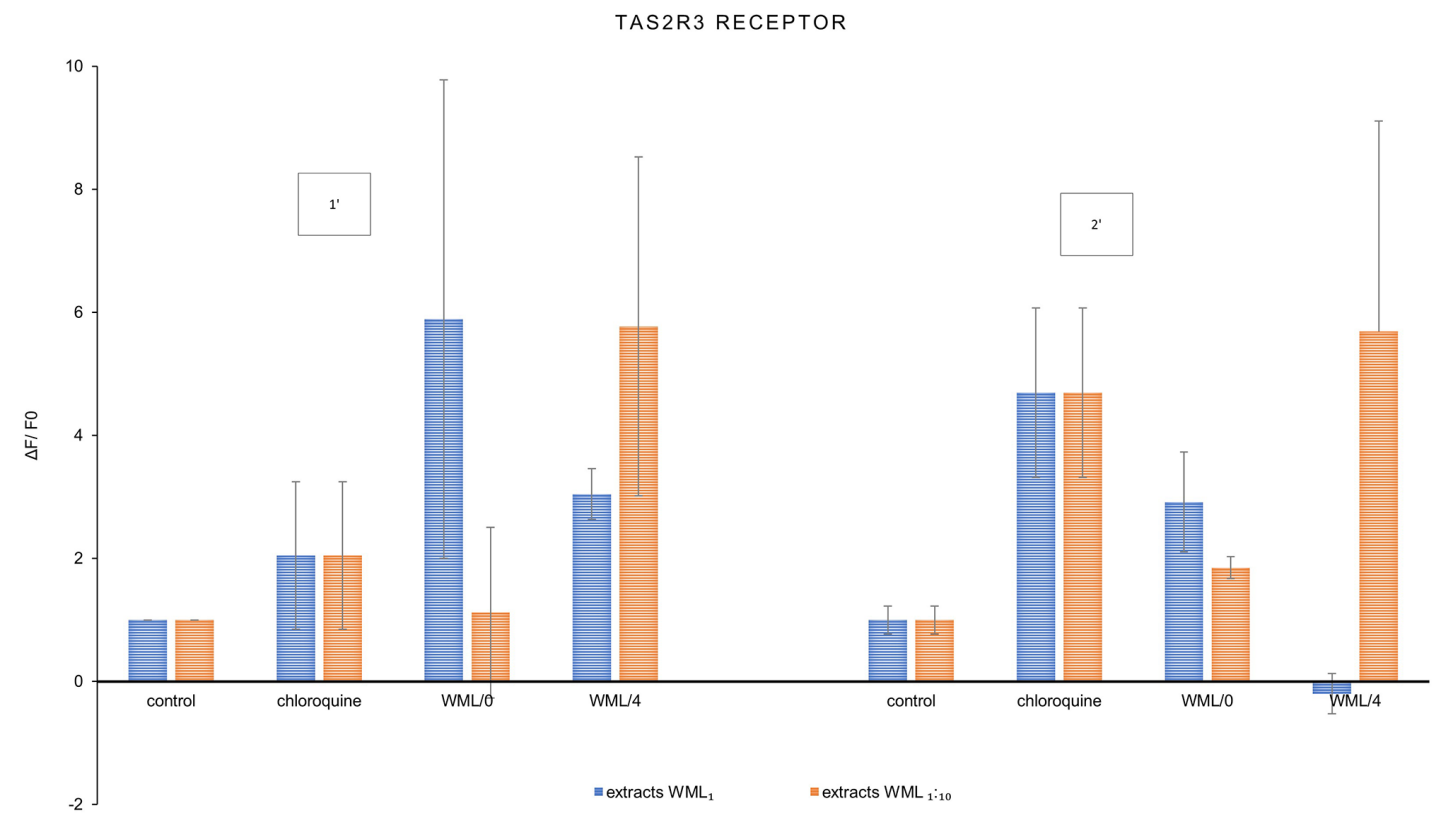
Changes in fluorescence (ΔF/F0) as a response of the TAS2R3 bitter taste receptor induced by *Morus alba* L. leaf extracts (WML_1_—not diluted, WML_1:10_—diluted 10-fold) and reference denatonium after 1 min [1’] and 2 min [2’] of interaction; n = 3

The key aim of this study was to observe the response of sweet (TAS1R2/ TAS1R3) and bitter (TAS2R13, TAS2R3) taste receptors to the *Morus alba* leaf extract. The results are summarized in the graphs below (Figs. 1, 2 and 3). The final results of the experiment were presented as the ratio of the increase in fluorescence of cells exposed to the tested extracts (not diluted WML_1_, diluted WML_1:10_) with reference to the control sample.

The study used bitter taste receptors type 2 which belong to a subgroup of G protein-coupled receptors (GPCRs) of family A. They are crucial in the perception of bitterness, and are increasingly used for therapeutic purposes. These chemosensory GPCR receptors are found in the plasma membrane of type II taste receptor cells (TRCs) in the taste buds of the tongue, pharynx, larynx, soft palate, and several other tissues throughout the body such as the intestinal epithelium (in enterocytes, tuft cells, cup cells, Paneth cells, microfollicular cells) [21].

The final product of the entire project was the identification of a substance that adequately modulates (preferably enhances) sweet taste, which would enhance the functional activation effect for sucralose at least several times. As an added value, it was assumed that such a substance from a natural source could simultaneously inhibit (reduce) the perception of bitter taste.

The study showed that from both WML variants, which were selected based on the authors’ previous research [3], conditioned leaves (WML/4) the closest to achieving the basic criterion in their effect on TSA1R2/TSA1R3 sweet taste receptors. At basal concentration (WML/4_1_) they enhanced sweet taste over three times (3.35-fold), and at 10-fold dilution (WML/4_1:10_)—3.8-fold, compared to sucralose, after 1 min of interaction. A similar effect was observed after 2 min of interaction, but to a lesser extent (Fig. 1).

Simultaneously, it was observed that conditioned mulberry leaf extract (WML/4) deactivated the bitter receptor TAS2R13, both after 1 and 2 min (Fig. 2), but enhanced bitter taste in the TAS2R3 receptor (stronger in samples WML_1:10_) (Fig. 3).

Ligands from WML/0 samples also enhanced sweet taste, stronger after 2 min of interaction with TSA1R2/TSA1R3 than those from the WML/4 sample (Fig. 1). When comparing both bitter taste receptors, mulberry extracts activated TAS2R3 (with chloroquine as positive) receptor stronger than TAS2R13 (with denatonium as positive).

In the context of the above results, it is worth bearing in mind that powdered mulberry leaf extracts themselves have a relatively bitter taste. However, the substances they contain react with taste receptors only to some extent. The food industry points to the bitter taste of the leaves precisely as a major problem in their industrial processing. This bitter taste of mulberry leaves is difficult to eliminate even as a result of technological processing. Despite some similarities, the mechanism of bitterness formation in mulberry leaves is quite different from that in tea leaves. It was shown that its intensity is a result of the interaction of many health-promoting compounds (amino acids, flavonoids, phenolic acids, alkaloids) on the one hand, and sugar alcohol metabolites on the other hand [22].

A study similar to the one presented here was conducted by Szczepaniak et al. [23] where authors recorded several times lower effects against receptors for the cornelian cherry than the white mulberry leaves analyzed. This raises hopes that in this area white mulberry leaves provide an interesting solution for the palatability of food products.

### Stability of Phenolic Acids and Flavonols from *Morus alba* L. Leaves during in vitro Digestion Process

According to the research model, the digestion process of WML/0 and WML/4 was simulated, imitating the human digestive tract. The digested samples were collected sequentially at six stages (from the stomach to the large intestine) of the process and analyzed qualitatively and quantitatively for the content of phenolic acids and flavonols, as presented in Table (Supplementary Material 3) and Figures (Supplementary Material 4).

The gastrointestinal tract plays the role of a gateway to the human body for ingredients obtained from the meal. Given that taste receptors have been identified in the extraoral area of the gastrointestinal tract, tasting the stability of bioactive compounds after the digestion process of white mulberry can determine the real strength of this effect. After all, the presence of receptors plays a key role in the protection of the human body, including the induction of appropriate motor and secretory responses, and the elimination of dangerous products (vomiting, diarrhea, temporary anorexia) [21].

Quantitative changes in different sections of the simulated gastrointestinal tract were evident for phenolic acids present in the model. With reference to retention times and UV-VIS spectra, ten phenolic acids were identified. Gallic acid predominated at all stages of the digestion process, while chlorogenic acid together with caffeic acid were also found in quite high amounts. Thus, this confirmed the previously identified relationships for phenolic acids in the characterization of the leaves of the analyzed Polish variety of white mulberry [3].

The content of each phenolic acid was determined to be statistically higher in WML/0 than in WML/4 samples. Moreover, the quantitative changes in these compounds were more intense in the case of WML/0. The WML/ 0 variant also showed highest total phenolic acid content. This was evident at stages A, B, C, and D, where, relative to analogous digested WML/4 samples, 2–4 times as many phenolic acids were recorded.

The in vitro digestion process resulted in fairly regular changes in the amounts of flavonols. Starting from the small intestine stage (D or E), the amounts were gradually reduced, the most at the terminal portion of the large intestine. For many flavonols, the decreasing content over the course of the process was preceded by an initial increase in their content at the A-D sections. Among the eight identified flavonols rutin and quercetin 3-(6-malonyl)-glucoside were found in the highest amounts at the beginning of the digestion process. In addition, smaller amounts of isoquercitrin, astragalin and myricetin were found. The amounts of quercetin, kaempferol and isorhamnetin were at levels lower than 1 µg*ml^− 1^ at all stages of digestion. The smallest variations in content between stages were recorded for isorhamnetin and quercetin (WML/0). Leaves conditioned for 4 h (WML/4) also showed the lowest content of each flavonol, and the total amount of flavonol was almost three times lower compared with WML/4 samples. The greatest quantitative losses of total flavonols were observed at stage F.

The observed different trends (increase or decrease) in the content of individual polyphenols at the initial stages of the simulated digestion process are supported by the literature. Some studies have pointed to the loss of polyphenols under oral and gastric conditions [24], while in others, authors have emphasized the stability of compounds during the passage through these stages. This was explained by too short exposure of the samples to the acidic environment of the stomach, which did not involve hydrolysis and release of polyphenols from cellular structures. In addition, it is speculated that low pH has a protective effect on polyphenol structures [25, 26].

After incubation under conditions simulating the stomach, the intestinal stage was simulated in a bioreactor. At the duodenal stage, pancreatic extract and bile acid salts were administered while increasing the alkalinity of the environment. A further pH increase to 7.4 corresponded to conditions in the small intestine, while holding at a pH 8.0 for 18 h—to conditions in the large intestine. The greatest reduction in phenolic acids and flavonols was observed after the F stage (large intestine). Polyphenols when reaching the colon are intensively transformed into smaller forms by the intestinal microflora. Their presence can also affect the growth of major strains of intestinal bacteria [27, 28]. It is increasingly emphasized that the antioxidant capacity of plant-derived foods is evidenced not only by polyphenol content, but also by the activity of phenolic metabolites of bacterial origin, high concentrations of which are recorded precisely within the colon [29].

Polyphenols are generally poorly absorbed during digestion, as they are converted through the action of digestive enzymes and intestinal microflora to lower molecular weight compounds. Animal and human studies showed the same relationship for many polyphenols, including chlorogenic acid, caffeic acid, ferulic acid and rutin [30].

## Conclusions

Most of the research that can be found is mainly focused on improving the quality of leaves or introducing them into the diet to achieve the best nutritional effect. Previous research on white mulberry leaves conducted by the present authors mainly focused on finding the best processing conditions for obtaining highly nutritional preparations. However, it turned out that a long conditioning process (4 h) resulted in the strongest reduction in the amount of bioactive compounds. It was therefore decided to find another, sustainable way of utilization of this preparation. Fortunately, this work found a new direction for its usage - as a taste modulator. It seems that the approach taken towards such preparation of mulberry leaves may have broad technological perspectives. From the practical point of view, it is believed that consumers who are sensitive to bitter taste, especially children or the elderly, could be interested in products with reduced or even removed bitter taste when improving or changing their diet. Moreover, the same problem is problem is observed for the intake of some dietary supplements or even medicines (bitter taste may discourage their use). New functional foods are very often enriched with components less familiar to the average consumer, quite exotic, and with a perculiar taste. Therefore, white mulberry leaves (their addition to the recipes of new products instead of synthetic sweeteners) are proposed as the natural solution to the above technological problems and to support such consumers at the molecular level in the future. On the other hand, safety verification is required before obtaining food additive status—starting with an analysis of toxic metabolites of fungi, microbiology tests, amounts of metals and polycyclic aromatic hydrocarbons, and ending with the cell line, animal and finally human studies. To the best of the authors knowledge, no studies have been published to date that identify white mulberry leaves as modulators of taste receptors. Thus, their practical use in this area may prove to be new and valuable to the functional food industry after further research on this plant.

### Electronic Supplementary Material

Below is the link to the electronic supplementary material.

Supplementary Material 1

Supplementary Material 2

Supplementary Material 3

Supplementary Material 4

## Data Availability

The datasets of the current study are available from the corresponding author on reasonable request.
