# Supplementary material for: Morus alba L. Leaves (WML) Modulate Sweet (TAS1R) and Bitter (TAS2R) Taste in the Studies on Human Receptors – A New Perspective on the Utilization of White Mulberry Leaves in Food Production?
Source: Plant Foods Hum Nutr. 2023 Oct 5;78(4):748–54. doi: 10.1007/s11130-023-01107-0 (PMC10665252; doi:10.1007/s11130-023-01107-0)
Supplement: Supplementary file 1 — Supplementary Material 1 [file 11130_2023_1107_MOESM1_ESM.doc]

**Supplementary Material 2.** Materials and Methods.

**Plant Material and Reagents**

The main plant material was white mulberry (*Morus alba* L.) leaves (WML), Polish var. żółwińska wielkolistna (WML) picked manually in June and July 2011 from a mulberry tree farm (Institute of Natural Fibres & Medicinal Plants, Pętkowo, central Poland) (DMS: 52º 12' 13.448" N 17º 15' 8.607" E).

Standards for phenolic acids (gallic acid, protocatechuic acid, 4-hydroxybenzoic acid, vanillic acid, caffeic acid, chlorogenic acid, syringic acid, *p*-coumaric acid, ferulic acid, sinapic acid), and flavonols (rutin, isoquercitrin, quercetin 3-O-(6”-O-malonyl)-β-D-glucoside, astragalin, myricetin, quercetin, kaempferol, isorhamnetin) were purchased from Sigma Aldrich, Germany. Reagents for receptors test were purchased from: IDT, Belgium (sequence coding G16-gust44), Invitrogen USA (pcDNA 3.1 vector), Biowest, USA (DMEM, Gibco, USA (Gltamax, PenStrep, sodium pyruvate), VWR, Poland (FBS), GenScript, USA, Sigma Aldrich, ATCC, USA (HEK293T, TAS2R13, TAS2R3). Other chemicals were of analytical grade or chromatographic grade, purchased from POCH, Gliwice, Poland, or Merck, Darmstadt, Germany.

**Morus Alba Leaves – Conditioning Process**

WML were processed at the Institute of Agricultural and Food Biotechnology in Poznań, Poland (IAFB) on a semi-technological scale. The fresh, whole leaves were 1) shredded with a chopper-crusher (Stihl, Viking GE 103, Tengen, Germany) into small fragments (ca. 50–70 mm x 10–20 mm), 2) divided into batches, 3) placed on wooden sieve trays in prisms (100 cm long, 40 cm wide, 10–15 cm high), and 4) conditioned for 4 hours (industrial dryer - SSO, Izoterma, Łany, Poland) at a temperature of 32.0˚C ± 3.0 (WML/4). One part of the chopped leaves was not conditioned (sample: WML/0). Next, each part of WML was air-dried (inlet:90˚C, outlet: 60˚C) in a tunnel dryer (IAFB,, Poland), and grounded (400 rpm, 15 s, mill - Retsch, GM200, Haan, Germany) to 0.8–1.25 mm powder (measured through a set of sieves). Then, the leaves were used in the digestion process.

**Extract Preparation**

The following water extraction method was used according to Przeor et al. [19]: 10.0 g ± 0.2 of WML was extracted two times for 15 min with boiling distilled water (1^st^-100 mL, 2^nd^- 40 mL) (EM, Thermo Scientific, Waltham, MA, USA), Both parts were separated from the sediment, filtered, and pooled into one sample. The extract was used in receptor analyses.

**Effect of Morus Alba Leaves on Receptors**

Two extracts were used in the study: 1) the water extract of WML (WML_1_), and 2) the water extract of WML diluted with PBS buffer (10-fold) (WML_1:10_) (Biowest, USA). The analysis was based on three steps according to Szczepaniak et al. [21]:

| Step | Description | |
| --- | --- | --- |
| 1 | Transfection with TAS2R13 and TAS2R3 genes | Synthetic sequence, coding G16-gust44 (IDT, Belgium) - amplification matrix;  insert into the pcDNA 3.1 vector (Invitrogen, USA); transfection of HEK293T cells; cell cultures dissolved in supplemented DMEM medium (Biowest, USA); incubation for 3 weeks; transfection with the lipofection method with genes coding bitter taste receptors (TAS2R13, TAS2R3) (GenScript, USA); separation with electrophoresis. |
| 2 | Transfection with TAS1R genes | Genes coding sweet taste receptors (TAS1R2/TAS1R3) cloned into the PSF-CMV-CMV-SBFI-UB-PURO—DUAL CMV vector (Sigma Aldrich, Poland), transfection to the HEK293T cell (ATCC, USA) with the lipofection method. |
| 3 | Calcium release test | Release of Ca^2+^ determined by using the Fluo-4 Direct™ Calcium Assay Kit (ThermoFisher) and then fluorescence measurement (Spectra-Max i3x, Molecular Devices, USA); results were normalized with the results of the MTT test each time after the experiment; positive control samples: 10 mM of sucralose for TAS1R2/TAS1R3, 30 mM of chloroquine for TAS2R3, 30 mM of denatonium for TAS2R13. |

The result was presented as ΔF/ F0 (fluorescence growth of cells with WML / intensity of fluorescence of control sample).

**Digestion Process**

A simulation of the digestion process was carried out using a static *in vitro* digestion model (Table 2).

Table 2. Schedule of conditions stages occurring at the in vitro digestion simulation.

| Digestion stage | Actor | Action time |
| --- | --- | --- |
| stomach | Pepsin (Sigma-Aldrich, Germany) | 2 hours |
| duodenum | Pancreatic extract +  bile acids (Sigma-Aldrich, Germany) | 0.5 hour |
| small intenstine | Intenstine microflora in BHI (Merck, Germany) | 2 hours |
| large intenstine |  | 18 hours |
| pH adjustment | 1M HCl, 1M NaOH (Merck, Germany) | |
| Aseptic conditions | - autoclave (121˚C, 15 min, Sanyo Techno Solutions Tottorico sterilizer, Japan) - laminar chamber (Safe 2020, ThermoScientific) - 72% ethanol solution (POCH, Poland) | |

A 1 dm^3^ Biostat B Plus laboratory bioreactor (Sartorius Stedim Biotech) equipped with an Easy Ferm plus PHI K8 160 electrode (Hamilton) was used. Intestinal microflora (30 ml, incubated at 37˚C for 24 hours prior to administration), distributed in BHI (Merck KGaA, Germany) was used to simulate conditions in the intestinal track. Digested samples of WML were stored as frozen.

**Content of Phenolic Acids and Flavonols**

The content of phenolic acids and flavonols in the samples was determined using the HPLC/DAD technique. Separation was performed on Agilent Technology's Zorbax SB C18 column (150 mm x 3.9 mm ID, 5 µm) based on the method described by Przeor et al. [3].Detection of separated phenolic acids was performed at λ=260 nm: gallic acid, protocatechuic acid, 4-hydroxybenzoic acid, vanillic acid, caffeic acid, and at λ=310 nm: chlorogenic acid, syringic acid, *p*-coumaric acid, ferulic acid, sinapic acid. Flavonols were detected at λ=370 nm: rutin, isoquercitrin, quercetin 3-O-(6”-O-malonyl)-β-D-glucoside, astragalin, myricetin, quercetin, kaempferol, isorhamnetin. Tests were performed in triplicates. The external standard method was used for quantitative assay (Table 3).

Table 3. Chromatographic conditions used in HPLC/DAD analysis.

|  | Wavelength | | Compound | | | Standard curve |
| --- | --- | --- | --- | --- | --- | --- |
| Phenolic acids | λ=260 nm | | gallic acid | | | y= 54.013x+143.85 |
|  |  |  | protocatechuic acid | | | y= 191.07x+82.424 |
|  |  |  | 4-hydroxybenzoic acid | | | y= 301.61x+15.069 |
|  |  |  | vanillic acid | | | y= 188.56x+9.42 |
|  |  |  | caffeic acid | | | y= 48.406x+11.419 |
|  | λ=310 nm | | chlorogenic acid | | | y= 277.55x+51.95 |
|  |  |  | syringic acid | | | y= 107.48x+23.398 |
|  |  |  | *p*-coumaric acid | | | y= 428.009x+2.376 |
|  |  |  | ferulic acid | | | y= 270.86x+14.292 |
|  |  |  | sinapic acid | | | y= 227.16x+12.951 |
| Flavonols | λ=370 nm | | rutin | | | y= 0.656x-1.096 |
|  |  |  | isoquercitrin | | | y= 1.099x+0.929 |
|  |  |  | quercetin 3-O-(6”-O-malonyl)-β-D-glucoside | | | y= 0.794x+0.952 |
|  |  |  | astragalin | | | y= 0.901x-1.744 |
|  |  |  | myricetin | | | y= 1.685x-4.267 |
|  |  |  | quercetin | | | y= 2.121x-8.568 |
|  |  |  | kempferol | | | y= 2.284x-6.032 |
|  |  |  | isorhamnetin | | | y= 2.317x-24.833 |
| Accuracy of the method | | | | 96.99 - 101.95% | | |
| LOD | | | | 10 ng*ml^-1^ | | |
| LOQ | | | | 3.3 ng*ml^-1^ | | |
| Solvents | | A: H_2_O : H_3_PO_4_, pH=2,7 | | | B: H_2_O : CH_3_CN, (v/v, 1:1) | |
| Duration of analysis | | 76 min | | | | |
| Sample preparation | | Each digested sample was filtrate through a syringe filter with 0.45 μm pore size. | | | | |
| Sample injection | | 20 µl | | | | |
| Column temperature | | 20˚C ± 0,5 | | | | |
| Gradient | | The method started with 90% solvent A, decreased to 56% at t= 63 min and returned to 90% at t= 76 min. | | | | |
| Device | | Agilent Infinity 1290, with diode array detector. | | | | |
