# Supplementary material for: Morus alba L. Leaves (WML) Modulate Sweet (TAS1R) and Bitter (TAS2R) Taste in the Studies on Human Receptors – A New Perspective on the Utilization of White Mulberry Leaves in Food Production?
Source: Plant Foods Hum Nutr. 2023 Oct 5;78(4):748–54. doi: 10.1007/s11130-023-01107-0 (PMC10665252; doi:10.1007/s11130-023-01107-0)
Supplement: Supplementary file 3 — Supplementary Material 3 [file 11130_2023_1107_MOESM3_ESM.doc]

**Supplementary Material 3.** Average amount (± standard deviation) of flavonols and phenolic acids in white mulberry leaves (digested samples) at each step of *in vitro* digestion.

| Compound, amount | | WML/0 | | | | | | | | | | | | | | | | | WML/4 | | | | | | | | | | | | | | | | | | |
| --- | --- | --- | --- | --- | --- | --- | --- | --- | --- | --- | --- | --- | --- | --- | --- | --- | --- | --- | --- | --- | --- | --- | --- | --- | --- | --- | --- | --- | --- | --- | --- | --- | --- | --- | --- | --- | --- |
|  |  | 0 | A | | | B | | | C | | | D | | | E | | | F | 0 | A | | | B | | | C | | | D | | | E | | | F | |  |
| RUT ◾ | [μg * ml^-1^] | - | 103.88^b^ | | 100.08^b^ | | | 84.95^b^ | | | 76.84^b^ | | | 51.01^b^ | | | 0.68^a^ | | - | 33.39^a^ | | 39.12^a^ | | | 25.91^a^ | | | 26.83^a^ | | | 33.60^a^ | | | 11.51^b^ | | |  |
|  |  | - | ±0.39 | | ±0.16 | | | ±4.80 | | | ±0.24 | | | ±0.49 | | | ±0.11 | | - | ±0.49 | | ±0.24 | | | ±0.03 | | | ±0.82 | | | ±4.46 | | | ±0.19 | | |  |
|  | [μg *mg^-1^ WML] | 0.28 | 6.49 | | 6.26 | | | 5.31 | | | 4.81 | | | 3.19 | | | 0.04 | | 0.42 | 2.09 | | 2.45 | | | 1.62 | | | 1.68 | | | 2.10 | | | 0.72 | | |  |
|  |  | ±0.05 | ±0.02 | | ±0.01 | | | ±0.30 | | | ±0.05 | | | ±0.09 | | | ±0.02 | | ±0.08 | ±0.07 | | ±0.04 | | | ±0.01 | | | ±0.08 | | | ±0.09 | | | ±0.4 | | |  |
| ISQ ◾ | [μg * ml^-1^] | - | 32.60^b^ | | 29.91^b^ | | | 30.33 ^b^ | | | 32.30^b^ | | | 21.09^b^ | | | 1.28^a^ | | - | 11.00^a^ | | 14.63^a^ | | | 11.58^a^ | | | 12.25^a^ | | | 16.99^a^ | | | 18.39^b^ | | |  |
|  |  | - | ±0.93 | | ±1.64 | | | ± 2.18 | | | ±1.32 | | | ±1.30 | | | ±0.55 | | - | ±0.09 | | ±0.03 | | | ±0.03 | | | ±0.04 | | | ±1.34 | | | ±0.19 | | |  |
|  | [μg *mg^-1^ WML] | 0.21 | 2.04 | | 1.87 | | | 1.89 | | | 2.02 | | | 1.32 | | | 0.08 | | 0.29 | 0.69 | | 0.91 | | | 0.72 | | | 0.77 | | | 1.06 | | | 1.15 | | |  |
|  |  | ±0.07 | ±0.12 | ±0.07 | | | ±0.11 | | | ±0.11 | | | ±0.09 | | | ±0.00 | | | ±0.04 | ±0.05 | ±0.04 | | | ±0.03 | | | ±0.13 | | | ±0.08 | | | ±0.05 | | |  | |
| MAL  ◾ | [μg * ml^-1^] | - | 82.12^b^ | 71.73^b^ | | | 83.36 ^b^ | | | 92.47^b^ | | | 61.34^b^ | | | 41.88^b^ | | | - | 30.61^a^ | 37.39^a^ | | | 31.26^a^ | | | 35.29^a^ | | | 44.18^a^ | | | 24.75^a^ | | |  | |
|  |  | - | ±0.21 | ±0.06 | | | ± 1.81 | | | ±1.11 | | | ±2.90 | | | ±0.15 | | | - | ±0.07 | ±0.06 | | | ±0.01 | | | ±0.04 | | | ±1.27 | | | ±0.14 | | |  | |
|  | [μg *mg^-1^ WML] | 0.21 | 5.13 | 4.48 | | | 5.21 | | | 5.78 | | | 3.83 | | | 2.62 | | | 0.16 | 1.91 | 2.34 | | | 1.95 | | | 2.21 | | | 2.76 | | | 1.55 | | |  | |
|  |  | ±0.08 | ±0.08 | ±0.10 | | | ±0.14 | | | ±0.07 | | | ±0.02 | | | ±0.06 | | | ±0.01 | ±0.05 | ±0.04 | | | ±0.09 | | | ±0.14 | | | ±0.18 | | | ±0.11 | | |  | |
| AST ◾ | [μg * ml^-1^] | - | 11.98^b^ | 11.10^b^ | | | 11.20^b^ | | | 12.87^b^ | | | 6.93^b^ | | | 5.07^a^ | | | - | 4.58^a^ | 6.23^a^ | | | 5.06^a^ | | | 5.14^a^ | | | 4.79^a^ | | | 6.86^b^ | | |  | |
|  |  | - | ±0.16 | ±0.08 | | | ±2.31 | | | ±0.66 | | | ±0.54 | | | ±0.16 | | | - | ±0.12 | ±0.01 | | | ± 0.01 | | | ±0.04 | | | ±0.10 | | | ±0.09 | | |  | |
|  | [μg *mg^-1^ WML] | 0.20 | 0.75 | 0.69 | | | 0.70 | | | 0.80 | | | 0.43 | | | 0.32 | | | 0.31 | 0.29 | 0.39 | | | 0.32 | | | 0.32 | | | 0.30 | | | 0.43 | | |  | |
|  |  | ±0.07 | ±0.05 | ±0.06 | | | ±0.08 | | | ±0.16 | | | ±0.19 | | | ±0.13 | | | ±0.08 | ±0.11 | ±0.12 | | | ±0.08 | | | ±0.07 | | | ±0.06 | | | ±0.02 | | |  | |
| MYR ◾ | [μg * ml^-1^] | - | 13.92^b^ | 13.90^b^ | | | 15.00^b^ | | | 19.86^b^ | | | 15.70^b^ | | | 1.61^b^ | | | - | 5.35^a^ | 6.44^a^ | | | 5.49 ^a^ | | | 6.26^a^ | | | 8.00^a^ | | | 2.43^a^ | | |  | |
|  |  | - | ±0.09 | ±0.08 | | | ±3.19 | | | ±0.63 | | | ±3.11 | | | ±0.31 | | | - | ± 0.09 | ±0.01 | | | ± 0.01 | | | ±0.00 | | | ±1.01 | | | ±0.96 | | |  | |
|  | [μg *mg^-1^ WML] | 0.06 | 0.87 | 0.87 | | | 0.94 | | | 1.24 | | | 0.98 | | | 0.10 | | | 0.09 | 0.34 | 0.40 | | | 0.34 | | | 0.39 | | | 0.50 | | | 0.15 | | |  | |
|  |  | ±0.01 | ±0.16 | ±0.14 | | | ±0.09 | | | ±0.11 | | | ±0.10 | | | ±0.02 | | | ±0.01 | ±0.11 | ±0.13 | | | ±0.04 | | | ±0.05 | | | ±0.06 | | | ±0.08 | | |  | |
| QUE ◾ | [μg * ml^-1^] | - | 0.33^b^ | 0.30^a^ | | | 0.37^b^ | | | 0.82^b^ | | | 0.81^b^ | | | 0.63^b^ | | | - | 0.26^a^ | 0.31^a^ | | | 0.33 ^a^ | | | 0.27^a^ | | | 0.32^a^ | | | 0.58^a^ | | |  | |
|  |  | - | ±0.00 | ±0.00 | | | ±0.07 | | | ±0.02 | | | ±0.06 | | | ±0.03 | | | - | ±0.00 | ±0.01 | | | ± 0.00 | | | ±0.00 | | | ±0.02 | | | ±0.02 | | |  | |
|  | [μg *mg^-1^ WML] | 0.03 | 0.02 | 0.02 | | | 0.02 | | | 0.05 | | | 0.05 | | | 0.04 | | | 0.06 | 0.02 | 0.02 | | | 0.02 | | | 0.02 | | | 0.02 | | | 0.04 | | |  | |
|  |  | ±0.00 | ±0.00 | ±0.00 | | | ±0.01 | | | ±0.01 | | | ±0.01 | | | ±0.01 | | | ±0.01 | ±0.01 | ±0.00 | | | ±0.00 | | | ±0.01 | | | ±0.00 | | | ±0.01 | | |  | |
| KEM ◾ | [μg * ml^-1^] | - | 0.18^a^ | 0.17^a^ | | | 0.19^a^ | | | 0.32^b^ | | | 0.41^b^ | | | 0.16^a^ | | | - | 0.16^a^ | 0.17^a^ | | | 0.19 ^a^ | | | 0.16^a^ | | | 0.17^a^ | | | 0.20^b^ | | |  | |
|  |  | - | ±0.02 | ±0.00 | | | ±0.02 | | | ±0.03 | | | ±0.09 | | | ±0.01 | | | - | ±0.00 | ±0.00 | | | ± 0.00 | | | ±0.00 | | | ±0.01 | | | ±0.01 | | |  | |
|  | [μg *mg^-1^ WML] | 0.01 | 0.01 | 0.01 | | | 0.01 | | | 0.02 | | | 0.03 | | | 0.01 | | | 0.01 | 0.01 | 0.01 | | | 0.01 | | | 0.01 | | | 0.01 | | | 0.01 | | |  | |
|  |  | ±0.00 | ±0.01 | ±0.00 | | | ±0.00 | | | ±0.00 | | | ±0.00 | | | ±0.00 | | | ±0.00 | ±0.00 | ±0.00 | | | ±0.00 | | | ±0.01 | | | ±0.01 | | | ±0.01 | | |  | |
| ISR ◾ | [μg * ml^-1^] | - | 0.58^a^ | 0.58^a^ | | | 0.59^a^ | | | 0.60^b^ | | | 0.58^b^ | | | 0.58^a^ | | | - | 0.58^a^ | 0.59^a^ | | | 0.57 ^a^ | | | 0.56^a^ | | | 0.55^a^ | | | 0.55^a^ | | |  | |
|  |  | - | ±0.04 | ±0.00 | | | ±0.01 | | | ±0.01 | | | ±0.01 | | | ±0.01 | | | - | ±0.00 | ±0.00 | | | ± 0.00 | | | ±0.00 | | | ±0.00 | | | ±0.02 | | |  | |
|  | [μg *mg^-1^ WML] | 0.00 | 0.04 | 0.04 | | | 0.04 | | | 0.04 | | | 0.04 | | | 0.04 | | | 0.00 | 0.04 | 0.04 | | | 0.04 | | | 0.04 | | | 0.04 | | | 0.03 | | |  | |
|  |  | ±0.00 | ±0.01 | ±0.01 | | | ±0.01 | | | ±0.01 | | | ±0.01 | | | ±0.01 | | | ±0.00 | ±0.02 | ±0.01 | | | ±0.01 | | | ±0.01 | | | ±0.01 | | | ±0.00 | | |  | |
| GAL ◾ | [μg * ml^-1^] | - | 7.96^b^ | 7.35^b^ | | | 7.42^b^ | | | 7.32^b^ | | | 4.59^b^ | | | 2.72^a^ | | | - | 2.43^a^ | 3.29^a^ | | | 2.79 ^a^ | | | 3.09^a^ | | | 3.09^a^ | | | 4.2^b^ | | |  | |
|  |  | - | ±0.03 | ±0.07 | | | ±0.02 | | | ±0.00 | | | ±0.01 | | | ±0.02 | | | - | ±0.00 | ±0.07 | | | ± 0.01 | | | ±0.02 | | | ±0.02 | | | ±0.01 | | |  | |
|  | [μg *mg^-1^ WML] | 0.19 | 0.50 | 0.46 | | | 0.46 | | | 0.46 | | | 0.29 | | | 0.17 | | | 0.20 | 0.15 | 0.21 | | | 0.18 | | | 0.19 | | | 0.19 | | | 0.26 | | |  | |
|  |  | ±0.04 | ±0.08 | ±0.09 | | | ±0.07 | | | ±0.12 | | | ±0.07 | | | ±0.13 | | | ±0.08 | ±0.07 | ±0.04 | | | ±0.08 | | | ±0.10 | | | ±0.07 | | | ±0.06 | | |  | |
| PRO ◾ | [μg * ml^-1^] | - | 0.59^b^ | 0.54^b^ | | | 0.46^b^ | | | 0.41^b^ | | | 0.35^b^ | | | 0.13^b^ | | | - | 0.23^a^ | 0.28^a^ | | | 0.20 ^a^ | | | 0.22^a^ | | | 0.20^a^ | | | 0.05^a^ | | |  | |
|  |  | - | ±0.00 | ±0.00 | | | ±0.00 | | | ±0.00 | | | ±0.00 | | | ±0.00 | | | - | ±0.00 | ±0.00 | | | ± 0.00 | | | ±0.00 | | | ±0.00 | | | ±0.00 | | |  | |
|  | [μg *mg^-1^ WML] | 0.14 | 0.04 | 0.03 | | | 0.03 | | | 0.03 | | | 0.02 | | | 0.01 | | | 0.22 | 0.01 | 0.02 | | | 0.01 | | | 0.01 | | | 0.01 | | | 0.01 | | |  | |
|  |  | ±0.05 | ±0.03 | ±0.00 | | | ±0.02 | | | ±0.01 | | | ±0.01 | | | ±0.01 | | | ±0.07 | ±0.00 | ±0.01 | | | ±0.00 | | | ±0.00 | | | ±0.00 | | | ±0.00 | | |  | |
| HYD ◾ | [μg * ml^-1^] | - | 0.03^b^ | 0.04^a^ | | | 0.03^b^ | | | 0.04^b^ | | | 0.03^b^ | | | 0.08^b^ | | | - | 0.02^a^ | 0.03^a^ | | | 0.02 ^a^ | | | 0.02^a^ | | | 0.02^a^ | | | 0.01^a^ | | |  | |
|  |  | - | ±0.00 | ±0.00 | | | ±0.00 | | | ±0.00 | | | ±0.00 | | | ±0.00 | | | - | ±0.00 | ±0.00 | | | ± 0.00 | | | ±0.00 | | | ±0.00 | | | ±0.00 | | |  | |
|  | [μg *mg^-1^ WML] | 0.01 | 0.01 | 0.01 | | | 0.01 | | | 0.01 | | | 0.01 | | | 0.01 | | | 0.13 | 0.01 | 0.01 | | | 0.01 | | | 0.01 | | | 0.01 | | | 0.01 | | |  | |
|  |  | ±0.00 | ±0.00 | ±0.00 | | | ±0.00 | | | ±0.00 | | | ±0.00 | | | ±0.00 | | | ±0.08 | ±0.00 | ±0.00 | | | ±0.00 | | | ±0.00 | | | ±0.00 | | | ±0.00 | | |  | |
| VAN  ◾ | [μg * ml^-1^] | - | 0.03^b^ | 0.04^b^ | | | 0.05^b^ | | | 0.07^b^ | | | 0.07^b^ | | | 0.09^b^ | | | - | 0.02^a^ | 0.02^a^ | | | 0.02 ^a^ | | | 0.02^a^ | | | 0.02^a^ | | | 0.01^a^ | | |  | |
|  |  | - | ±0.00 | ±0.00 | | | ±0.00 | | | ±0.00 | | | ±0.00 | | | ±0.00 | | | - | ±0.00 | ±0.00 | | | ± 0.00 | | | ±0.00 | | | ±0.00 | | | ±0.00 | | |  | |
|  | [μg *mg^-1^ WML] | 0.16 | 0.01 | 0.01 | | | 0.01 | | | 0.01 | | | 0.01 | | | 0.01 | | | 0.31 | 0.01 | 0.01 | | | 0.01 | | | 0.01 | | | 0.01 | | | 0.00 | | |  | |
|  |  | ±0.04 | ±0.00 | ±0.00 | | | ±0.00 | | | ±0.00 | | | ±0.00 | | | ±0.00 | | | ±0.09 | ±0.00 | ±0.01 | | | ±0.00 | | | ±0.00 | | | ±0.01 | | | ±0.00 | | |  | |
| CHL ◾ | [μg * ml^-1^] | - | 2.56^b^ | 2.40^b^ | | | 1.68^b^ | | | 1.41^b^ | | | 0.55^a^ | | | 0.00^a^ | | | - | 0.71^a^ | 0.98^a^ | | | 0.77 ^a^ | | | 0.72^a^ | | | 0.62^b^ | | | 1.07^b^ | | |  | |
|  |  | - | ±0.02 | ±0.01 | | | ±0.55 | | | ±0.01 | | | ±0.00 | | | ±0.00 | | | - | ±0.00 | ±0.00 | | | ± 0.24 | | | ±0.01 | | | ±0.00 | | | ±0.00 | | |  | |
|  | [μg *mg^-1^ WML] | 0.38 | 0.16 | 0.15 | | | 0.11 | | | 0.09 | | | 0.03 | | | 0.00 | | | 0.16 | 0.04 | 0.06 | | | 0.05 | | | 0.05 | | | 0.04 | | | 0.06 | | |  | |
|  |  | ±0.011 | ±0.07 | ±0.03 | | | ±0.08 | | | ±0.03 | | | ±0.01 | | | ±0.00 | | | ±0.04 | ±0.01 | ±0.01 | | | ±0.00 | | | ±0.01 | | | ±0.01 | | | ±0.01 | | |  | |
| CAF ◾ | [μg * ml^-1^] | - | 2.18^b^ | 1.88^b^ | | | 1.80^b^ | | | 1.40^b^ | | | 0.78^b^ | | | 0.58^a^ | | | - | 0.60^a^ | 0.83^a^ | | | 0.55 ^a^ | | | 0.63^a^ | | | 0.56^a^ | | | 0.97^b^ | | |  | |
|  |  | - | ±0.02 | ±0.00 | | | ±0.03 | | | ±0.00 | | | ±0.00 | | | ±0.00 | | | - | ±0.00 | ±0.00 | | | ± 0.00 | | | ±0.00 | | | ±0.00 | | | ±0.00 | | |  | |
|  | [μg *mg^-1^ WML] | 0.38 | 0.14 | 0.12 | | | 0.11 | | | 0.09 | | | 0.05 | | | 0.04 | | | 0.16 | 0.04 | 0.05 | | | 0.03 | | | 0.04 | | | 0.04 | | | 0.06 | | |  | |
|  |  | ±0.07 | ±0.03 | ±0.04 | | | ±0.01 | | | ±0.02 | | | ±0.01 | | | ±0.00 | | | ±0.06 | ±0.01 | ±0.02 | | | ±0.01 | | | ±0.01 | | | ±0.00 | | | ±0.02 | | |  | |
| SYR ◾ | [μg * ml^-1^] | - | 0.04^b^ | 0.03^b^ | | | 0.02^b^ | | | 0.01^a^ | | | 0.00^a^ | | | 0.00^a^ | | | - | 0.02^a^ | 0.02^a^ | | | 0.01 ^a^ | | | 0.01^a^ | | | 0.00^a^ | | | 0.00^a^ | | |  | |
|  |  | - | ±0.00 | ±0.00 | | | ±0.00 | | | ±0.00 | | | ±0.00 | | | ±0.00 | | | - | ±0.00 | ±0.00 | | | ± 0.00 | | | ±0.00 | | | ±0.00 | | | ±0.00 | | |  | |
|  | [μg *mg^-1^ WML] | 0.02 | 0.01 | 0.01 | | | 0.01 | | | 0.01 | | | 0.00 | | | 0.00 | | | 0.01 | 0.01 | 0.01 | | | 0.00 | | | 0.00 | | | 0.00 | | | 0.00 | | |  | |
|  |  | ±0.00 | ±0.00 | ±0.00 | | | ±0.00 | | | ±0.00 | | | ±0.00 | | | ±0.00 | | | ±0.00 | ±0.00 | ±0.00 | | | ±0.00 | | | ±0.00 | | | ±0.00 | | | ±0.00 | | |  | |
| CUM ◾ | [μg * ml^-1^] | - | 0.01^a^ | 0.01^a^ | | | 0.01^b^ | | | 0.01^b^ | | | 0.01^b^ | | | 0.00^b^ | | | - | 0.01^a^ | 0.01^a^ | | | 0.00 ^a^ | | | 0.00^a^ | | | 0.00^a^ | | | 0.00^a^ | | |  | |
|  |  | - | ±0.00 | ±0.00 | | | ±0.00 | | | ±0.00 | | | ±0.00 | | | ±0.00 | | | - | ±0.00 | ±0.00 | | | ± 0.00 | | | ±0.00 | | | ±0.00 | | | ±0.00 | | |  | |
|  | [μg *mg^-1^ WML] | 0.05 | 0.00 | 0.00 | | | 0.01 | | | 0.00 | | | 0.00 | | | 0.01 | | | 0.10 | 0.00 | 0.00 | | | 0.00 | | | 0.00 | | | 0.00 | | | 0.00 | | |  | |
|  |  | ±0.01 | ±0.01 | ±0.00 | | | ±0.01 | | | ±0.00 | | | ±0.00 | | | ±0.00 | | | ±0.02 | ±0.00 | ±0.00 | | | ±0.00 | | | ±0.00 | | | ±0.00 | | | ±0.00 | | |  | |
| FER ◾ | [μg * ml^-1^] | - | 0.03^b^ | 0.03^b^ | | | 0.03^b^ | | | 0.03^b^ | | | 0.02^b^ | | | 0.01^b^ | | | - | 0.01^a^ | 0.01^a^ | | | 0.01 ^a^ | | | 0.01^a^ | | | 0.01^a^ | | | 0.01^a^ | | |  | |
|  |  | - | ±0.00 | ±0.00 | | | ±0.00 | | | ±0.00 | | | ±0.00 | | | ±0.00 | | | - | ±0.00 | ±0.00 | | | ± 0.00 | | | ±0.00 | | | ±0.00 | | | ±0.00 | | |  | |
|  | [μg *mg^-1^ WML] | 0.14 | 0.01 | 0.01 | | | 0.01 | | | 0.01 | | | 0.01 | | | 0.01 | | | 0.16 | 0.01 | 0.01 | | | 0.01 | | | 0.01 | | | 0.01 | | | 0.01 | | |  | |
|  |  | ±0.02 | ±0.01 | ±0.01 | | | ±0.01 | | | ±0.01 | | | ±0.00 | | | ±0.00 | | | ±0.03 | ±0.01 | ±0.01 | | | ±0.01 | | | ±0.01 | | | ±0.01 | | | ±0.01 | | |  | |
| SIN ◾ | [μg * ml^-1^] | - | 0.02^b^ | 0.01^a^ | | | 0.01^a^ | | | 0.02^b^ | | | 0.01^b^ | | | 0.00^b^ | | | - | 0.01^a^ | 0.01^a^ | | | 0.01 ^a^ | | | 0.01^a^ | | | 0.00^a^ | | | 0.00^a^ | | |  | |
|  |  | - | ±0.00 | ±0.00 | | | ±0.00 | | | ±0.00 | | | ±0.00 | | | ±0.00 | | | - | ±0.00 | ±0.00 | | | ± 0.00 | | | ±0.00 | | | ±0.00 | | | ±0.00 | | |  | |
|  | [μg *mg^-1^ WML] | 0.21 | 0.01 | 0.01 | | | 0.01 | | | 0.01 | | | 0.01 | | | 0.00 | | | 0.31 | 0.01 | 0.01 | | | 0.01 | | | 0.00 | | | 0.00 | | | 0.00 | | |  | |
|  |  | ±0.03 | ±0.00 | ±0.00 | | | ±0.00 | | | ±0.00 | | | ±0.00 | | | ±0.00 | | | ±0.12 | ±0.00 | ±0.00 | | | ±0.00 | | | ±0.00 | | | ±0.00 | | | ±0.00 | | |  | |

WML – white mulberry leaves; WML/0 – non-conditoned white mulberry leaves; WML/4 – conditoned white mulberry leaves; RUT- rutin; ISQ – isoquercitrin; MAL – quercetin 3-O-(6”-O-malonyl)-β-D-glucoside; AST – astragalin; MYR – myricetin; QUE – quercetin; KEM – kaempferol; ISR – isorhamnetin; GAL – gallic acid; PRO – protocatechuic acid; HYD – 4-hydroxybenzoic acid ; VAN – vanillic acid; CHL – chlorogenic acid; CAF – caffeic acid; SYR – syringic acid; CUM – *p*-coumaric acid; FER – ferullic acid; SIN – sinapic acid; A – initial stage in stomach; B – final stage in stomach; C – stage in duodenum (pH 6.0); D – stage in small intestine (pH 7.4); E – initial stage in large intestine; F – final stage in large intestine; ^a, b^ – different letters indicate statistically significant differences in a two-factor analysis of variance (ANOVA).
