# Supplementary material for: Morus alba L. Leaves (WML) Modulate Sweet (TAS1R) and Bitter (TAS2R) Taste in the Studies on Human Receptors – A New Perspective on the Utilization of White Mulberry Leaves in Food Production?
Source: Plant Foods Hum Nutr. 2023 Oct 5;78(4):748–54. doi: 10.1007/s11130-023-01107-0 (PMC10665252; doi:10.1007/s11130-023-01107-0)

**Supplementary Material 4.** Changes in phenolic acids and flavonols content during simulated digestion process.


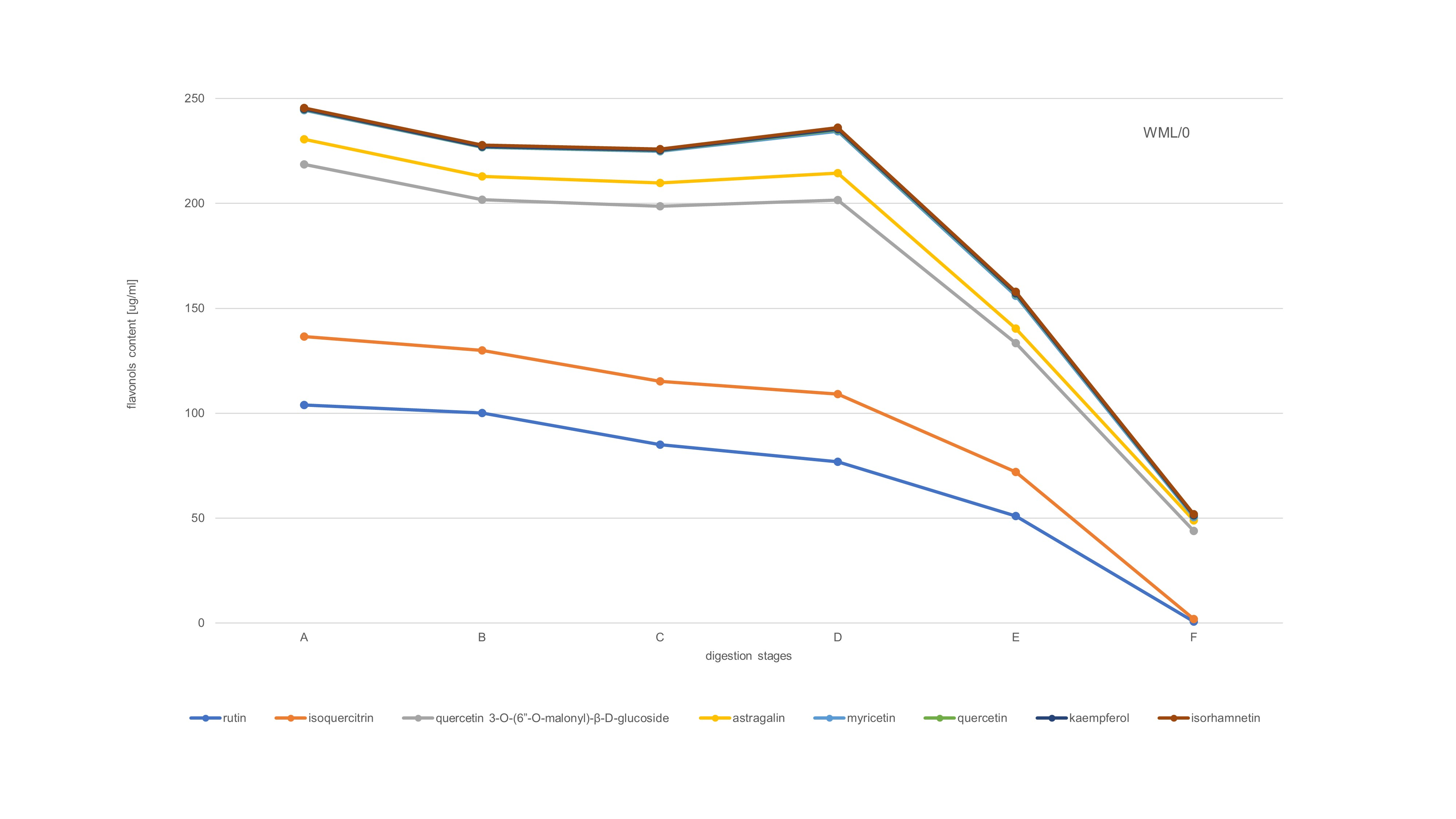


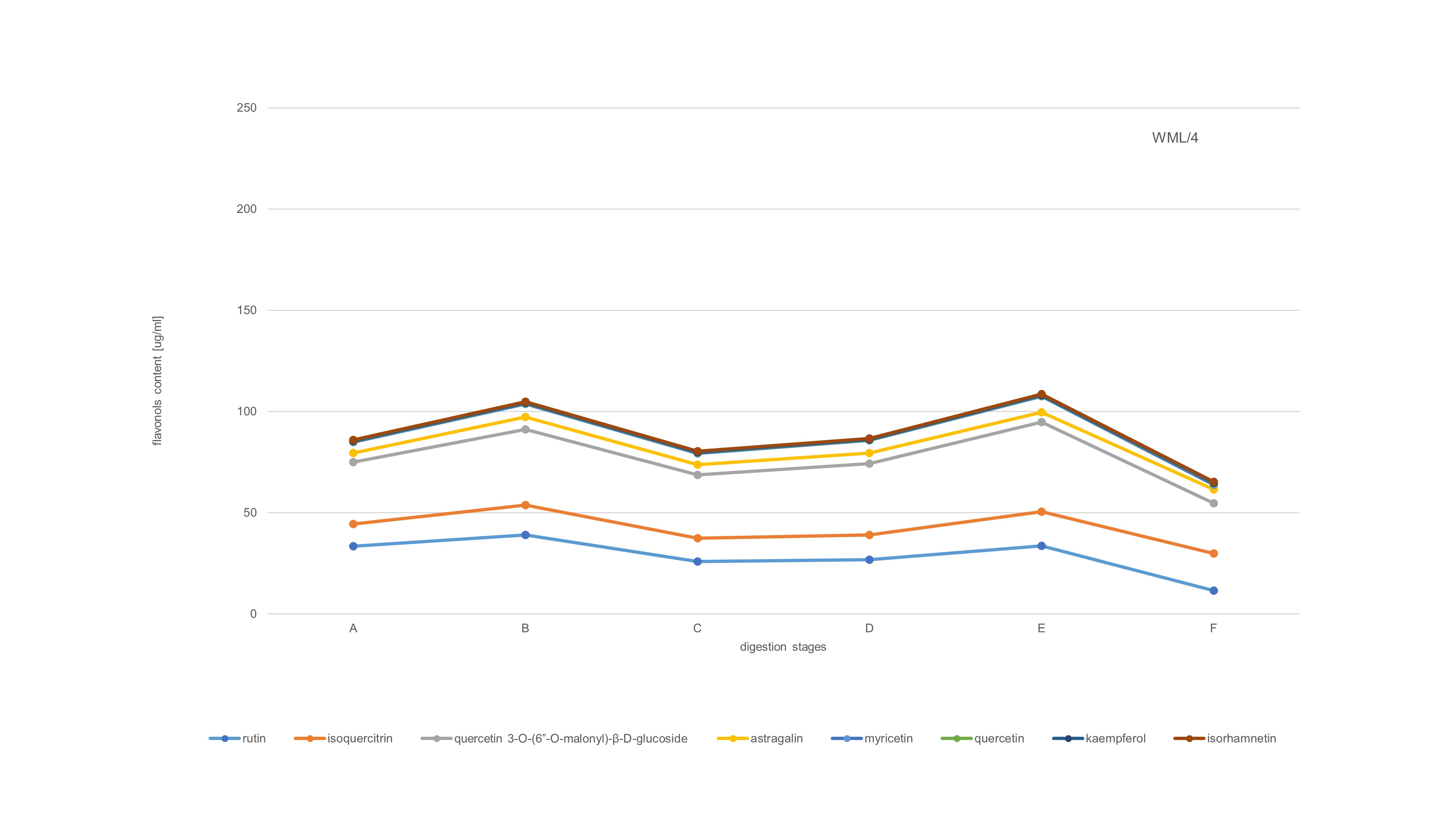


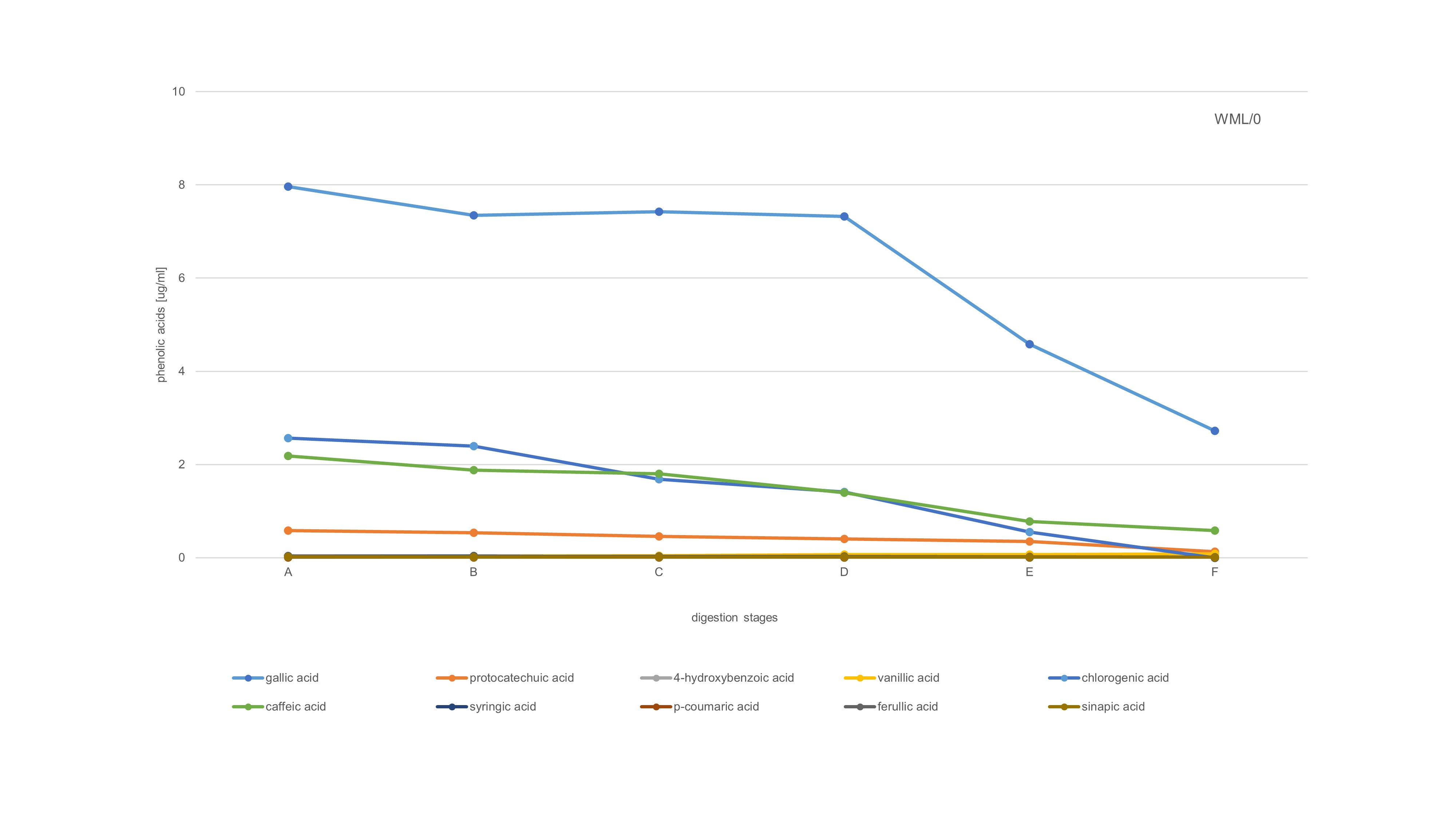


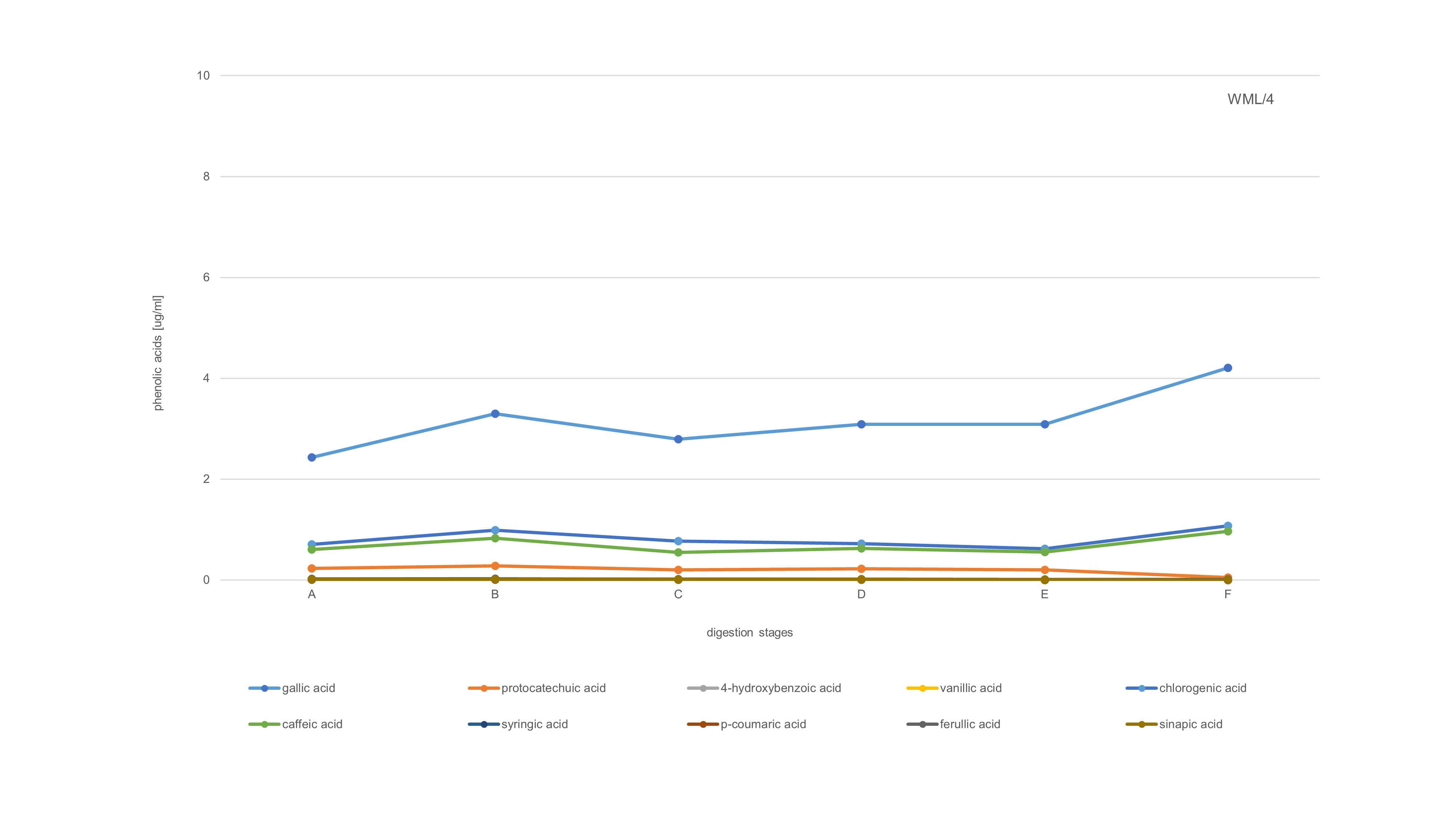

Supplement: Supplementary file 4 — Supplementary Material 4 [file 11130_2023_1107_MOESM4_ESM.doc]
